# Inflammation in Parkinson’s disease: role of glucocorticoids

**DOI:** 10.3389/fnana.2015.00032

**Published:** 2015-04-02

**Authors:** María-Trinidad Herrero, Cristina Estrada, Layal Maatouk, Sheela Vyas

**Affiliations:** ^1^Clinical and Experimental Neuroscience (NiCE-IMIB), Institute for Bio-Health Research of Murcia, School of Medicine, Campus Mare Nostrum, University of MurciaMurcia, Spain; ^2^Laboratory of Gene Regulation and Adaptive Behaviors, Department of Neuroscience Paris Seine, INSERM U 1130, CNRS UMR 8246, UPMC UM 119, Université Pierre et Marie CurieParis, France

**Keywords:** glucocorticoid receptor, Parkinson’s disease (PD), neuroinflammation, neurodegeneration, microglia

## Abstract

Chronic inflammation is a major characteristic feature of Parkinson’s disease (PD). Studies in PD patients show evidence of augmented levels of potent pro-inflammatory molecules e.g., TNF-α, iNOS, IL-1β whereas in experimental Parkinsonism it has been consistently demonstrated that dopaminergic neurons are particularly vulnerable to activated glia releasing these toxic factors. Recent genetic studies point to the role of immune system in the etiology of PD, thus in combination with environmental factors, both peripheral and CNS-mediated immune responses could play important roles in onset and progression of PD. Whereas microglia, astrocytes and infiltrating T cells are known to mediate chronic inflammation, the roles of other immune-competent cells are less well understood. Inflammation is a tightly controlled process. One major effector system of regulation is HPA axis. Glucocorticoids (GCs) released from adrenal glands upon stimulation of HPA axis, in response to either cell injury or presence of pathogen, activate their receptor, GR. GR regulates inflammation both through direct transcriptional action on target genes and by indirectly inhibiting transcriptional activities of transcriptional factors such as NF-κB, AP-1 or interferon regulatory factors. In PD patients, the HPA axis is unbalanced and the cortisol levels are significantly increased, implying a deregulation of GR function in immune cells. In experimental Parkinsonism, the activation of microglial GR has a crucial effect in diminishing microglial cell activation and reducing dopaminergic degeneration. Moreover, GCs are also known to regulate human brain vasculature as well as blood brain barrier (BBB) permeability, any dysfunction in their actions may influence infiltration of cytotoxic molecules resulting in increased vulnerability of dopamine neurons in PD. Overall, deregulation of glucocorticoid receptor actions is likely important in dopamine neuron degeneration through establishment of chronic inflammation.

## Introduction

Parkinson’s disease (PD) is a common age-related neurodegenerative disorder characterized by cardinal motor symptoms that include bradykinesia with resting tremor, rigidity and gait disturbance. These motor symptoms become evident when already 70–80% of nigrostriatal terminals have degenerated. At present, therapeutic treatments, for example, levodopa, mostly address motor symptoms. However, a wide spectrum of non-motor clinical features such as REM (Rapid eye movement) sleep disturbances, autonomic dysfunction, depression, anxiety, cognitive impairment or falling are associated with PD, and are moreover debilitating and unresponsive to dopamine-related treatments. Thus, PD is a complex systemic disorder with non-motor symptoms often preceding motor symptoms and worsening with disease progression (Berg et al., 2014; Goldman and Postuma, 2014). PD has an age-adjusted incidence of 13.5–13.9 per 100.000 person-years, and a prevalence of 315 per 100,000 individuals (the second most common worldwide). As PD affects predominantly older people, its prevalence increases with age from 428 at 60–69 years to 1,903 per 100.000 in 80 years old people (Abdullah et al., 2014; Pringsheim et al., 2014).

The major neuropathological hallmarks of PD are progressive degeneration of dopaminergic neurons in the Substantia Nigra pars compacta (SNpc); presence of proteinaceous inclusions called Lewy bodies (LBs) and chronic inflammation. However, the initial causes and underlying mechanisms pertaining to these neuropathological features in the majority of patients, classified as sporadic PD, remain unknown. Recent studies indicate that combined genetic, environmental factors and aging confer risk for developing sporadic PD rather than genetic or environmental factor individually. Approximately 5–10% of PD patients present the familial form of the disease with either autosomal dominant or recessive mode of inheritance. Epidemiological analysis confirm that up to 40% of PD patients with age at onset of less than 30 years and 17% of those with age at onset of less than 50 years will probably present the familial form of the disease. At least 18 loci as well as 12 genes with Mendelian inheritance and highly penetrant mutations causing rare monogenic forms have been identified. The genetic discovery of point mutations, duplication or triplication of *SNCA* (synuclein) gene coding for α-synuclein protein (reviewed in Goedert et al., 2013) with demonstration by Spillantini et al. (1997) that α-synuclein is a major component of LBs, led Braak et al. (2003) to staging PD according to appearance of α-synuclein containing LBs and Lewy neuritis with disease severity. Accordingly to Braak’s hypothesis PD progresses in neuronally-connected ascending manner to dorsal motor nucleus of the glossopharyngeal and vagus nerves likely from gut and/or olfactory mucosa (stage 1 and 2), then from lower brain stem to midbrain including nigral regions (stage 3 and 4) and lastly to the neocortical regions (stage 5 and 6). However, it has been suggested that LB pathology alone is not sufficient and that associated neuronal loss leads to Parkinsonism (Buchman et al., 2012).

In last few years, large genetic and genome-wide association (GWA) studies together with meta-analyses have led to significant and rapid advances in the genetic basis of sporadic PD, with realization both for its wide implication and complexity (Lill et al., 2012; Clarimón and Kulisevsky, 2013). There is great expectation for further insights with advent of new DNA sequencing technologies (exome and whole-genome sequencing) and the NeuroX genotyping platform (Nalls et al., 2015). These studies have identified at least 20 susceptibility loci as risk for sporadic PD (Nalls et al., 2014). As yet, the true significance of many of these loci is still unknown. Of interest, susceptibility related to *SNCA* and *LRRK2* (Leucine rich repeat kinase 2) loci consistently observed are also genes that have been identified in the monogenic, autosomal dominant familial PD patients. Occurrence of somatic mosaicism has been also hypothesized in the etiology of some cases of PD (Kim and Jeon, 2014). Cases of somatic mosaicism in many central nervous system (CNS) disorders have been reported, for example, somatic mutation in the *presenilin-1* gene associated with Alzheimer’s disease (Beck et al., 2004), in *SPG4/SPAST* (spastic paraplegia4/spastin) causing spastic paraplegia (Depienne et al., 2007) or *MECP2* (methyl CpG binding protein) resulting in Rett syndrome (Topçu et al., 2002). Thus far, no cases of PD with somatic mosaicism are known, as well, in this regard results of study on *SNCA* somatic mutations by Proukakis et al. (2014) were negative.

Summing up: (a) although at present there is a rapid progress and evolution in technology to unravel genetic basis of PD, most PD risk is not understood; (b) the pathogenicity arising from several of identified gene mutations remains to be determined; (c) highly penetrant gene mutations (as *DJ-1, LRRK2, Parkin, PINK1* (PTEN-induced putative kinase 1), and *SNCA*) cause rare monogenic forms of the disease; (d) somatic mosaicism could shed light on the heterogeneity of PD; and (e) additive mechanisms is suggested in risk for PD, increasing with the number of risk alleles carried by a single subject e.g., in HLA (human leucocyte antigen) region (Hill-Burns et al., 2011).

There is strong epidemiological evidence to show that aging is a single most important risk factor for PD, with increase in incidence between fifth to eight decades. Modifications that occur in specific brain regions during aging, such as increased oxidative and nitrative stress, changes in glial functions, dysfunction of proteasomes and lysosomes and altered α-synuclein protein are also manifestations of PD (Collier et al., 2011; Kieburtz and Wunderle, 2013). Environmental toxins identified as risk for PD are herbicides (e.g., paraquat or rotenone), heavy metals such as manganese and lead, nanoparticles as air pollutants, head trauma or well water. Thus, for example, it was shown that people exposed to pesticides and harboring Cytochrome P450 2D6 (CYP2D6) genotype with poor metabolic capacity for xenobiotics are at increased risk for developing PD (Elbaz et al., 2004). Epidemiologic link also exists between rotenone and PD. Rotenone is a powerful inhibitor of mitochondrial complex 1 and interestingly complex I deficiency is found in PD. The role of viral infections as risk factor has been evoked ever since the famous and controversial von Economo’s encephalitis lethargica pandemic suspected to be caused by H1N1 (Hemagglutinin1 neuraminidase1) influenza virus where patients exhibited Parkinsonism symptoms (Ravenholt and Foege, 1982). Recently, animals infected with highly pathogenic H5N1 virus showed clear motor deficits as well human cases with encephalitis have been reported (Jang et al., 2009). Epigenetic modifiers could be potential mediators of environmental factors (Portela and Esteller, 2010). Aberrant epigenetic modifications include changes in gene functions or gene expression but without changing the DNA sequence: non-coding RNA-mediated changes of gene expression, DNA methylation or post-transcriptional modifications and acetylation of histones (Nalls et al., 2014). For instance, the methylation of the Tumor Necrosis Factor alpha (TNF-α) promoter is significantly decreased in the SNpc of PD patients compared with controls or with the methylation in the cortex (Pieper et al., 2008) suggesting increased susceptibility of dopamine neurons to TNF-α mediated inflammation (Barcia et al., 2005, 2011).

Thus, aging together with genetic susceptibility and cumulative environmental factors such as air pollutants, pesticides, infections or exposure to heavy metals likely have a role in the development of idiopathic PD.

### Immune System and the Etiology of Parkinson’s Disease

All of the above environmental factors together with multiple cellular changes occurring during aging can impact immune functions. There is now a growing realization, particularly from genetic studies, that immune system is most likely involved in the etiology as well as early phases of PD, thus the inflammatory component of the disease may simply not be a consequence of neuronal dysfunction and neurodegeneration. In GWA studies, a number of susceptibility loci that have been identified as strong risk factors, are related to both innate and adaptive immune functions, for example, *HLA-DQB1*, *LRRK2*, *GPNMB* (glycoprotein NMB), or *BST-1* (bone marrow stromal cell antigen) (Liu et al., 2011; Pihlstrøm et al., 2013). In this regard, *LRRK2*, *Parkin*, *PLA2G6* (phospholipase A2, group VI), *DJ-1* and *SNCA* genes mutated in both familial and idiopathic PD are also known to function in microglia and astrocytes (Russo et al., 2014). Interestingly, several studies have identified risk of PD with polymorphisms present in the promoter regions of *IL-1β* and *TNF-α* genes that augment the expression of these genes and whose protein products have potent pro-inflammatory activity (Wahner et al., 2007). Moreover, polymorphisms reported in other pro-inflammatory genes e.g., CD14, *HLA-DBQ1*, *HLA-DRA*, HLA-DRB1, *HLA-DRB5* can also increase the risk for PD (Ahmed et al., 2012). In the analysis of potential markers of motor and cognitive progression, SNPrs 6482992 of clarin3 (CLRN3) was described as the best predictor of cognitive deterioration (Chung et al., 2012) whereas SNPrs 10958605 as involved in neuroinflammatory pathways (Cappellano et al., 2013). The implication of early involvement of immune system is also reinforced by epidemiological studies showing a prolonged use of NSAIDs (Nonsteroidal anti-inflammatory) particularly ibruprofen subsequently lowers the risk of PD (Rees et al., 2011).

### Neuroinflammation in PD

As a progressive neurodegenerative disorder, PD is a multifactorial complex disease most likely evolving because of the genetic and environmental risk factors, as well as cellular alterations and aging. Inflammatory component in PD not only encompasses deregulation of inflammatory pathways resulting from genetic vulnerability but also immune alterations associated with aging and with primary activation of glia in the face of neuronal injury. Aging affects the functions of immune system, resulting in so-called “immune senescence”. Specifically, advancing age has been associated with chronic mild inflammation in the SNpc, thereby rendering dopaminergic neurons vulnerable to degeneration (Kanaan et al., 2010). Increasing evidence points to the role of active peripheral inflammation in PD that can contribute to the initiation and/or the progression of the disease by, for example, exacerbating and synergizing with the discordant central inflammatory response to drive dopaminergic neurodegeneration. Combination of aging, heritable risk factors and exposure to environmental agents has been suggested as potential host-pathogen specific pathophysiologic elements that can cause deregulation of both innate and adaptive immune system responses (Kanaan et al., 2008; Chao et al., 2014). Thus, in both sporadic and familial PD, immune activation occurring at multiple levels would play an important role in PD pathology.

Evidence of an on-going neuroinflammation in affected brain regions in PD stems from analyses of pro-inflammatory cytokines (Interferon gamma, IFN-γ; TNF-α; Interleukin-6, IL-6; or Interleukin-1β, IL-1β) showing their accumulation in both cerebrospinal fluid and post-mortem brain (Mogi et al., 1994; Dobbs et al., 1999; Reale et al., 2009a,b). Recently, it has been demonstrated that the serum levels of IL-6 and the chemokine ligand 5 (CCL5) also known as Regulated on Activation, Normal T cell Expressed and Secreted (RANTES) were significantly increased in PD patients, and importantly, RANTES levels correlated with the severity and duration of the disease (Tang et al., 2014). Furthermore, the augmentation of iNOS (Inducible nitric oxide synthase) observed in SN and striatum of PD (Hunot et al., 1996) suggests that the toxicity originating both from cytokines/chemokines and inflammation-derived oxidative stress could contribute to dopaminergic neuronal degeneration and progression of the disease (Orr et al., 2005; Wilms et al., 2007). Numerous studies in experimental PD models indicate that dopamine neurons are particularly vulnerable to both oxidative stress and inflammatory attack (McGeer and McGeer, 2008; Pott Godoy et al., 2008). Interestingly, in this regard, Lipopolysaccharide (LPS)-activated microglia in the vicinity of dopamine neurons in SN induce degeneration of these neurons whilst sparing GABAergic and serotonergic neurons, suggesting a selective dopamine neuron vulnerability to inflammation (Liu and Bing, 2011).

Inflammation and immune-related responses may be viewed not only as determinant factors in disease progression but also as pathogenic processes in the onset of both familial and sporadic PD (Halliday and Stevens, 2011; Chao et al., 2014; Dzamko et al., 2014). On this point, presence of activated microglia, visualized by PET (positron emission tomography) analysis using radioligand ^11^C-PK-11195, was recently reported in the SN and putamen of PD patients diagnosed within a year from clinical onset (Iannaccone et al., 2013). This, together with study of Ouchi et al. (Ouchi et al., 2005) suggests a microglial-mediated inflammatory process in early stage of PD. Several lines of evidence also point to relevant actions of different PD-linked gene mutations e.g., *SNCA* or *LRRK2* in stimulating inflammatory responses through activation of microglia and astrocytes thereby participating directly in chronic PD progression (Gillardon et al., 2012; Moehle et al., 2012; Harms et al., 2013). Both central and peripheral inflammation occurs in the prodromal stage of PD, which thus sustains disease progression (Dzamko et al., 2014; Su and Federoff, 2014). Overall, accumulation of pathological α-synuclein in PD brain leads to neurodegeneration with T-cell infiltration, microglial activation and increased production of inflammatory cytokines and chemokines (Harms et al., 2013). The detection of T lymphocytes and activated microglia in the SN of Parkinsonian patients is striking because systemic immune cells have to penetrate several barriers in order to reach the brain parenchyma.

The CNS was considered as an immunologically privileged site because of the lack of lymphatic vessels, the absence of classical major histocompatibility complex (MHC) positive antigen presenting cells, and the presence of barriers as the tanycytic barrier around the circumventricular organs or the neurovascular unit of the BBB. The latter is composed of endothelial cells, pericytes and astrocytes and associated strong, tight junctions prevent the entry of immune cells into the brain parenchyma. BBB is a metabolic and physical barrier that separates the CNS from the peripheral circulation, actively allowing the transports of nutrients to the brain but limiting passive diffusion of blood-borne solutes. However, in aging and in PD, a BBB disruption has been described with loss of the barrier permeability leading to secondary leukocyte migration within the brain parenchyma, reactive gliosis and damage to neurons (Stolp and Dziegielewska, 2009; Cabezas et al., 2014). BBB dysfunction in PD favors an invasion of immune cells (and/or peripheral mediators and factors as toxins or elements of adaptive immunity) into the brain parenchyma that provokes a progressive and self-perpetuating degenerative process (Monahan et al., 2008). Additionally, it has been demonstrated that PD patients have increased permeability of the intestinal epithelial barrier (Forsyth et al., 2011) as well as chronic enteric/colonic inflammation (Devos et al., 2013). As proposed by Braak et al. (2003), an environmental pathogen can cross the monolayer of polarized epithelial cells (the intestinal epithelial barrier) (Sharkey and Savidge, 2014) and enter into the terminal axons of the submucosal plexus spreading to the medulla oblongata via the vagal preganglionic innervation of the gut (Hawkes et al., 2009). Moreover, brain injuries or systemic infections can induce systemic inflammatory responses that easily communicate with brain. Both Alzheimer’s disease and PD have been associated with both the HLA region (Ahmed et al., 2012; Wissemann et al., 2013), and with the production of autoantibodies (Maetzler et al., 2014) suggesting putative genetic susceptibility to inflammation that could initiate the neuronal dysfunction.

Microglia are the resident innate immune cells in the brain. Being only 5–15% of the total cells of the brain, microglia functions include tissue repair and cellular homeostasis after neuronal injury. Activated microglia produce neurotoxic molecules, for example, pro-inflammatory cytokines, chemokines, complement proteins or nitric oxide. Additionally, activated microglia acquire phagocytic properties and develop neuro-immune interactions involving the expression of surface molecules as CD200/CD200R, CD47/CD172a, CX3C chemokine ligand 1 and its receptor (CX3CL1/CX3CR) and the complement regulatory proteins, complement components C1q and C3 in order to eliminate cellular debris and damaged neurons by gliapses (Barcia et al., 2012). However, microglial responses can have neuroprotective as well as harmful consequences mainly if there is a continuous exposure to a pro-inflammatory environment with a persistent release of inflammatory mediators (Bardou et al., 2014) as activated microglia can still persist even years after the toxic insults (Barcia et al., 2004; Jackson-Lewis and Smeyne, 2005; Block et al., 2007). In fact, if as a defense mechanism of the organism, an inflammatory response starts and continues without control, a chronic persistent inflammation environment in the brain can result in tissue destruction and progressive neurodegeneration.

### Glucocorticoids, Inflammation and Parkinson’s Disease

Inflammation is normally a tightly regulated process that acts to prevent pathogen invasion as well as cellular injury, whilst at the same time enabling tissue repair. Several endogenous mechanisms act to regulate the immune cell functions, which are involved in triggering an inflammatory process. Among them, the steroid hormone, glucocorticoid, is a known major regulator of immune system and inflammation. Glucocorticoids (GCs) are one of the most potent and effective anti-inflammatory agents in clinical use ever since the isolation of cortisone and its clinical application in the early 1950s by the Nobel Prize winners Hench, Kendall and Reichstein (Hench et al., 1950; Reichstein, 1951).

GCs (cortisol in humans and corticosterone in rodents) are endogenous steroid hormones synthesized in adrenal glands and secreted into systemic circulation. The GC secretion occurs in ultradian pulsatile manner (Hellman et al., 1970; Veldhuis et al., 1989) and over-riding this pattern is acute GC rise in response to a stressor (psychogenic or physical e.g., tissue injury or pathogen invasion) whereby increased levels of GCs exert important adaptive actions in multiple tissues to restore homeostasis (Young et al., 2004; McEwen, 2007). Both ultradian/circadian and stress-evoked GC secretion is tightly controlled by various negative feedback mechanisms affecting each component of HPA axis, notably synthesis and release of corticotropin-releasing hormone (CRH) from the paraventricular nucleus (PVN) of the hypothalamus and adrenocorticotropic hormone (ACTH) from anterior pituitary. Any change in negative feedback loops will affect HPA axis, resulting in altered ultradian/circadian rhythm of GC release often with abnormally high basal GC levels, which in turn could lead to GC resistance.

Measurement of plasma cortisol in idiopathic PD patients has consistently shown significantly elevated levels compared to age-matched control subjects, and as well correlating with impulsive behaviors (Bellomo et al., 1991; Stypula et al., 1996; Hartmann et al., 1997; Charlett et al., 1998; Djamshidian et al., 2011; Ros-Bernal et al., 2011). The high cortisol levels seem unrelated to L-DOPA treatment or disease duration (Müller et al., 2007). Elevated cortisol levels are observed in many other neurodegenerative diseases including Alzheimer disease (Huang et al., 2009). In PD, however, the normally quiescent nocturnal cortisol secretory pattern is particularly affected (Hartmann et al., 1997) raising the question as to whether the circadian control of HPA axis by suprachiasmatic nucleus is altered. The underlying causes of HPA deregulation and whether or how it impacts PD pathology is presently not well understood. However, presence of LBs in both adrenal glands and hypothalamus in PD has been reported (Wakabayashi and Takahashi, 1997; Braak et al., 2006), which may imply a role of α-synuclein pathology in HPA axis deregulation. In addition to neuronal networks regulating HPA axis through feed back loops, cytokines liberated by peripheral immune cells can also stimulate HPA axis in several ways. Potent inflammatory cytokines (TNF-α, Il-1β and Il-6) can induce release of GCs by directly stimulating CRH synthesizing neurons of PVN or indirectly by stimulating production of prostaglandin E2 synthesis in perivascular cells (Ericsson et al., 1994; Kang et al., 2006; Serrats et al., 2010). In addition, IL-6 was shown to directly act on anterior pituitary cells as well as in adrenal glands, via its receptor, to stimulate the synthesis of ACTH and GC respectively (Zarković et al., 2008). Thus, deregulated immune responses with elevated levels of pro-inflammatory cytokins may lead to chronic activation of HPA axis.

Once secreted, GCs act on diverse physiological processes ranging from metabolism, immune responses to cognition and behavior. Their therapeutic potential, however, has limitations as chronic use with sustained high levels of GCs can result in serious side effects such as diabetes, obesity, dyslipidemia, hypertension, osteoporosis or behavioral anomalies. GCs clearly exert anti-inflammatory actions especially in an inflammatory setting, however, a number of recent studies indicate that they also exert pro-inflammatory responses, which are cell-type dependent. Thus, in response to acute stress resulting in increased GC levels, high levels of pro-inflammatory mediators such as IL-1β were found (Dhabhar, 2002; O’Connor et al., 2003; Sorrells et al., 2007). In a microarray study by Galon et al. (2002) on human mononuclear cells, dexamethasone treatment was found to induce the expression of several innate-immune related genes in addition to down-regulation of pro-inflammatory genes. It is believed that this opposing action of GCs “prepares” the immune system to respond rapidly to harmful stimulus and subsequently GCs act to down-regulate the immune response to restore homeostasis.

### Glucocorticoid Regulation of Inflammation through GR

In brain, GC signaling is mediated by almost ubiquitously expressed GRs (GRs) as well as mineralocorticoid receptors (MR) that have restricted expression in neurons. However, it should be noted that MR is also expressed in glia (Sierra et al., 2008). GR, a prototype member of nuclear receptor superfamily (designated as NR3C1 in nomenclature of nuclear receptor family) is a ligand-activated transcription factor, it can also exert non-genomic actions (Groeneweg et al., 2011). GR is a modular protein with an N-terminal transactivation domain, a C-terminal ligand binding domain (LBD) and a central Zinc fingers-containing DNA-binding domain (DBD) that recognizes a specific DNA sequence. The LBD is the high affinity binding site for cortisol and other ligands. In humans, two major isoforms of GR, hGRα and hGRβ arising from alternative splicing have been described (Zhou and Cidlowski, 2005) and they differ in their C-terminal ligand-binding domain such that hGRβ cannot bind to endogenous or synthetic GCs. Experimental evidence indicates that hGRβ is expressed at low levels and it antagonizes the transcriptional activity of hGRα thus acting as dominant negative inhibitor of hGRα. However, recent genome-wide microarray studies indicate that hGRβ also regulates gene transcription (Kino et al., 2009). Interestingly, reduction in hGRα:hGRβ ratio has been associated with behavioral and mood disorders such as depression and schizophrenia (Perlman et al., 2004; Matsubara et al., 2006). In addition, alternative translational initiation sites generating 8 different GR proteins both in mouse and humans have been described (Oakley and Cidlowski, 2013).

Recent evidence shows that pulsatile pattern of GC secretion is crucial to proper GR transcriptional activity, thus loss of GC oscillatory pattern can result in continuous transcription with abnormal protein accumulation or GR targeting inappropriate genes leading to undesirable outcomes. GR is normally inert in the cytoplasm, in association with complex of proteins including heat-shock chaperones (HSP90, HSP70, HSP40, HSP23) and immunophilins such as FKBP51 (FK506 binding protein51), FKBP52, CP44 and PP5 (Grad and Picard, 2007). GC binding to this complex results in conformational change in GR exposing a nuclear localization signal resulting in importin-mediated translocation through the nuclear pore to the nucleoplasm.

In the nucleus, GR regulates transcription of its target genes in multiple, complex ways as well in highly cell- and context-specific manner. The transcriptional activity of GR has been especially studied with respect to its actions on metabolism and regulation of immune responses in the periphery. Multitude of studies indicates that GCs through GR influence each stage of inflammatory response i.e., from initiation, effector to resolution phases of an inflammatory reaction. Inflammatory response is triggered by specific receptors in immune cells and in this regard toll-like receptors (TLRs) activation and intracellular signaling cascade is thus far best characterized (Kawai and Akira, 2010) resulting in activation of transcriptional factors such as Nuclear factor kappa B (NF-κB), Activator Protein 1 (AP-1) or interferon response factors (IRFs). Each TLR family member (from 1–13 in mouse; all expressed in microglia) recognizes specific molecular signature present in either pathogens (PAMPs-Pathogen Associated Molecular Patterns) or molecules released by injured cells called DAMPs (Damage-associated Molecular Patterns). GR is reported to regulate key components of TLR signaling e.g., transforming growth factor beta-activated kinase 1 (TAK1; Bhattacharyya et al., 2010).

The GR regulation of inflammation is a result of both its transcriptional stimulatory and repressive activity. Classically, GR stimulates transcription of genes that act to inhibit inflammation and conversely it inhibits transcription of pro-inflammatory genes. GR stimulates transcription as homodimer binding to specific cognate DNA sequence, GAGAACAnnnTGTTCT, called Glucocorticoid Responsive Elements (GREs) present in the promoter regions of its target genes. This transcriptional activity of GR requires the presence of chromatin modifiers (e.g., Nuclear receptor coactivator NCoA1), basal transcriptional machinery and co-factors (CREB binding protein CBP, p300) (Rosenfeld and Glass, 2001). This mode of transcriptional activity has been notably described for genes coding for proteins of metabolic pathways such as glucose-6-phosphotase, fatty acid synthase or tyrosine aminotransferase as well as anti-inflammatory genes e.g., (Nuclear factor of kappa light polypeptide gene enhancer in B-cells inhibitor (IκB-α), MAPK phosphatase (MPK-1), IL-4, IL-10 or annexin-1 (De Bosscher et al., 2003). GR can also bind to negative GREs (nGREs) to repress transcription, thus among the genes identified containing nGREs are CRH as well as ACTH receptor in adrenal glands (Dostert and Heinzel, 2004; Surjit et al., 2011). Importantly, with regards to inflammation, GR can also inhibit transcription by tethering (i.e., through protein-protein interactions) or modulating the activity of other transcriptional factors, for example NF-κB, AP1 or IRF (Chinenov et al., 2013). This action mediated by GR monomers has been particularly studied in peripheral immune cells involving inhibition of expression of powerful pro-inflammatory genes as well as resolution of inflammation. The cross talk between AP1, NF-κB and GR is well documented (De Bosscher et al., 2003). As it has pertinence in the effects of GR observed in microglia it will be briefly reiterated here.

AP1 is comprised of heterodimers of c-Fos (C-Fos, FosB, Fra1 and 2), Jun (c-N, B-Jun, D-Jun) as well as ATF (Activating transcription factor) families of transcription factors, which controls expression of many cytokines. AP1 activity is stimulated by MAPK cascade resulting in activation of c-Jun-N terminal kinase (JNK), which phosphorylates c-Jun. GR regulates AP1 activity by associating with Jun-Fos complex at AP1 DNA elements in promoter regions of genes, inducing a conformational change in the complex that is not functional (Diamond et al., 1990). Additionally, GR also stimulates transcription of MAPK-phosphatase 1 (MKP-1) by binding to GRE elements present in its promoter region (as mentioned above) resulting in MKP-1 termination of JNK phosphorylation activity on c-Jun.

NF-κB signaling in positive immune regulation has been thoroughly characterized especially in the periphery (Karin and Greten, 2005). NF-κB comprises of RelA (p65), RelB, c-Rel, NF-κB1 (p50/p105 subunits) and NF-κB2 (p50/p100) proteins and the transcriptionally active dimers identified are: p65/p50 (classic NF-κB), p65/p65, p65/c-Rel, RelB/p50, RelB/p52 and, of note, Rel domains of these proteins bind to DNA. The p65/p50 NF-κB protein is normally sequestered in the cytoplasm by IκB family of proteins. NF-κB translocates to nucleus following phosphorylation of IκB by IKK kinases followed by rapid degradation of IκB. Importantly, phosphorylation of the p65 subunit is important for NF-κB activation. This involves phosphorylation of Serine 27Kuro6 of p65 by protein kinase A (PKA) catalytic subunit in complex with NF-κB and IκB as well as by nuclear localized MAPK-activated mitogen- and stress-activated protein kinase 1 (MSK1) in the nucleus. Interestingly GR was shown to decrease the nuclear pool of MSK1 thus down regulating NF-κB activity (Beck et al., 2008). With regards to its interaction with NF-κB, it was shown that upon GR activation by GC, GR is acetylated. In the nucleus, GR is deacetylated by histone deacetylase (HDAC2) (Ito et al., 2006) before it can physically bind to p65 subunit of NF-κB, functioning as transcriptional antagonist. Another manner by which GR can terminate NF-κB activation is by directly stimulating transcription of IκB-α as mentioned above.

### Innate Immune Regulation by GRs in Microglia during Dopamine Neurodegeneration

In the CNS the role of endogenous GCs in regulating expression of pro-inflammatory cytokines such as IL-1β, TNF-α or IL-6 was shown originally following peripheral administration of LPS in adrenalectomized mice (Goujon et al., 1996). The finding that HPA axis is reactive to CNS inflammation triggered by an intrastriatal LPS injection was revealed through prior challenge with systemic LPS that resulted in rise in systemic corticosterone levels with concomitant and significant reductions in proinflammatory TNF-α, Monocyte chemoattractant protein MCP-1, IκBα transcripts in lesioned striatal/cortical region (Nadeau and Rivest, 2002). Interestingly in this paradigm, LPS does not trigger neuronal degeneration. However, neuronal death was observed by prior treatment with GR antagonist RU486 suggesting that GCs acting through GR prevent neuronal degeneration (Nadeau and Rivest, 2003). Recently, the role played by microglial GR in regulating neuronal survival in this intrastriatal model of LPS was shown conclusively in mice with selective inactivation of GR in microglia/macrophages, GR^LysMCre^ mice (Carrillo-de Sauvage et al., 2013). Inflammation triggered by low dose of LPS (1–2 µg) injection has negligible effect on striatal or cortical neurons (Carrillo-de Sauvage et al., 2013), however the same dose of LPS injection in substantia nigra causes specific loss of dopamine neurons (Castaño et al., 2002) indicating a selective vulnerability of dopamine neurons to microglial inflammatory response mediated by LPS-activated TLR4. However, the fact that endogenous GCs activating GRs in microglia are neuroprotective during LPS-induced inflammation in cortex/striatum but not in midbrain substantia nigra implies that their actions in microglia during TLR4 activation may be region-specific. In this regard, recently the concept of microglial heterogeneity with respect to their functional capabilities, for example LPS/TLR4 signaling, has been evoked (Noh et al., 2014).

**Figure 1:**
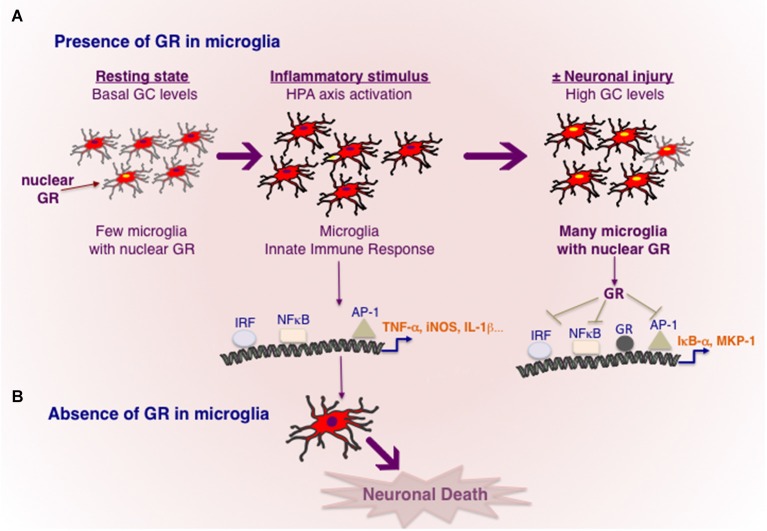
**Transcriptional regulation of inflammation by GR in microglia. (A)** In resting, healthy state without any cellular injury or pathogen invasion, GR is inactive in most microglia. Toxins e.g., MPTP or powerful inflammogen LPS would rapidly trigger innate immune response in microglia. In the case of LPS, TLR4 present in microglia results in activation of major transcription factors NF-κB, IRF and AP-1, known to orchestrate an inflammatory response. Cellular injury or pro-inflammatory cytokines would activate HPA axis and increased GC secretion. High circulating GC levels results in GR activation in microglia, which act to repress the transcriptional activity of NF-κB, IRF and AP-1 and also stimulating the expression of genes such as IκB-α and or MKP-1 known to inhibit NF-κB and AP-1 respectively. Neuronal injury and death are prevented. **(B)** In the absence of GR activity in microglia, microglia remain in activated state causing neuronal death.

Nigral dopamine neurodegeneration triggered by MPTP is significantly reduced by pharmacological treatments with GC agonists e.g., corticosterone that artificially increase GCs above endogenous levels, conversely adrenalectomy augments dopamine neuronal loss (Kurkowska-Jastrzebska et al., 2004; Sugama et al., 2009; Ros-Bernal et al., 2011) indicating that high levels of GCs present during MPTP intoxication protect dopamine neurons. Immuno-labeling of GR revealed its localization mainly in the nucleus of microglia and its quantification was carried out in substantia nigra and striatum in saline and MPTP injected mice. The results showed that number of microglia with nuclear GR augmented from 35% in resting state to 70–80% 3 days after MPTP injections, which then declined to almost normal levels after 3 weeks. Measurement of endogenous corticosterone levels showed a three-fold rise 1 day after MPTP (Ros-Bernal et al., 2011). Importantly, these results indicate that GR activation during endogenous rise in corticosterone levels is progressive concurring loss of dopamine neurons (Figure 1). However increasing GC levels by corticosterone treatment results in significant neuroprotection likely because GR activation in microglia is rapid enough to counteract the inflammatory response mounted by activated glia.

The precise actions of GR in microglia during dopamine neurodegeneration were studied using GR^LysMCre^ mice (Ros-Bernal et al., 2011). Functionally, absence of GR in microglia/macrophages resulted in significant dopamine neuronal loss in two paradigms of MPTP intoxication: (a) acute toxicity (4 injections/day) which is accompanied by intense microglial and astroglial activation of short duration; and (b) sub-chronic treatment (1 injection for 5 days) the loss of dopamine neurons is less, and morphologically, microglial activation is less apparent. In microglial GR mutant mice, both MPTP paradigms augmented microglial activation i.e., number hypertrophied microglia, compared to controls. Additionally, in sub-chronic paradigm, GR was found to prolong the duration of activation. Several molecules released by degenerating dopamine neurons can potentially trigger morphological and functional changes in microglia i.e., its activation status or its mobility (e.g., Matrix metalloprotease MMP-9, α-synuclein, Annese et al., 2015), however how the primary signals emitted from degenerating dopamine neurons trigger microglial activation is not well elucidated. With regards to regulation of inflammation, microglial GR was found to modulate 3 classes of inflammatory genes: (a) increasing expression of pro-inflammatory molecules in particular, TNF-α, iNOS, Intercellular adhesion molecule (ICAM); (b) anti-inflammatory genes e.g., MKP-1 (as described above for inhibiting AP1 transcriptional activity) and IL-1R2 which is a decoy receptor for IL-1 receptor1; (c) inflammatory caspases, i.e., caspases 1 and 4 as well as TLR3, TLR4, TLR9 and MyD88. The inflammatory caspases and TLRs are core components of innate immunity important for stimulating the transcriptional activity of AP1, NF-κB and IRF and thereby expression of plethora of inflammatory mediators. These findings indicate that GR not only inhibits the molecules like TNF-α known to execute the inflammatory reaction but also prevent excessive expression of upstream activators that initiate an inflammatory reaction.

Nuclear expression of p65 subunit of NF-κB, indicative of transcriptional activity NF-κB, was observed in microglia of SNpc in PD patients as well as in mice treated with MPTP. Moreover inhibiting NF-κB in mice significantly protected dopamine neurons against MPTP toxicity (Ghosh et al., 2007). Thus sustained transcriptional activity of NF-κB is likely involved in chronic activation of microglia in PD. Interestingly, GR was found to associate with p65 subunit of NF-κB in microglial cultures, as well in luciferase reporter assays GR inhibited its transcriptional activity (Ros-Bernal et al., 2011; Carrillo-de Sauvage et al., 2013). *In vivo*, Serine 276 phosphorylation of P65 subunit of NF-κB, indicative of its activation, was sustained in MPTP-lesioned SNpc and striatum of mutant GR microglial mice (Ros-Bernal et al., 2011).

The halting of inflammation is central to immune response. A failure to limit the amplitude and duration of this process as well as initiate a resolution phase can lead to chronic inflammatory state. In addition to GR, other members of nuclear receptor family e.g., Peroxisome proliferator activated receptor gamma (PPAR-γ), Liver X receptor (LXR), Estrogen receptor ER-β, Nuclear receptor NR4A family (Nurr 77, Nurr1) are expressed in microglia, thus they can also control microglial activation. In this regard, Nurr1 inhibition was found to increase NF-κB activity in glia resulting in exaggerated expression of pro-inflammatory mediators and increased loss of dopamine neurons following LPS injection in substantia nigra (Saijo et al., 2009).

**Figure 2:**
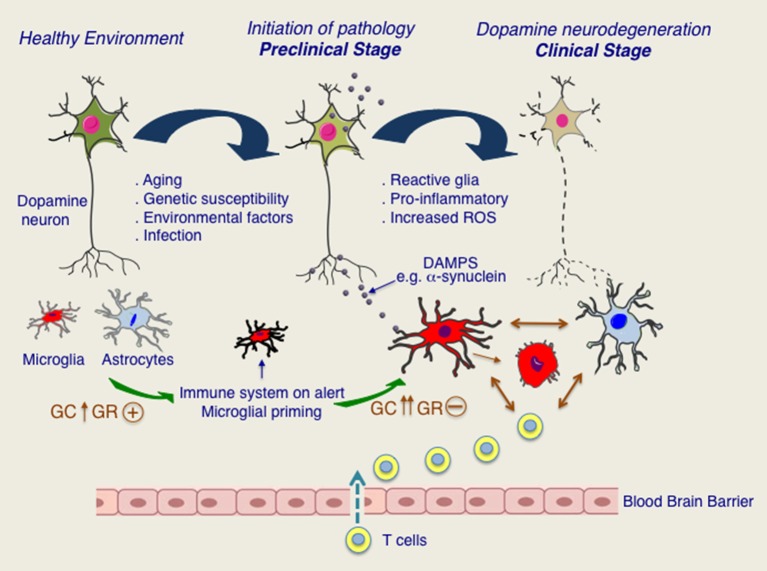
**Putative roles of glucocorticoids (GC) and glucocorticoid receptor (GR) in progression to chronic inflammation and dopamine neurodegeneration**. In healthy state, microglia and astroglia surrounding are quiescent. Aging as well as other stressors such as infections or PD-related genetic and environmental factors would put immune system on alert and possibly also stimulating HPA axis. Activation of HPA axis results in increase in GC levels and activation of GR. In pre-clinical stage, secretion of DAMPS, such as pathological form of α-synuclein would activate immune system as well as HPA axis. Persistent activation of HPA axis results in loss of its regulation and chronically high GC levels. Chronic GCs are known to result in GR dysfunction in immune cells. Microglia and astroglia remain activated creating a pro-inflammatory environment and augmenting oxidative stress. Disruption in blood brain barrier resulting in T cell infiltration further promotes glial activation. Dopamine degeneration is progressively increased leading to clinical manifestation of PD.

Regarding GCs, it is possible that in PD, GR functions in immune cells are compromised because of chronically elevated levels of cortisol. The putative scenario of dysfunction of GR signaling in PD is illustrated in Figure 2. Different stressors such as aging, infections, environmental and genetic susceptibility factors would activate HPA axis resulting in augmentation of circulating GC levels and activation of GR. In parallel, activation of peripheral immune system would result in increased circulating levels of pro-inflammatory molecules e.g., Il-1β known to activate HPA axis and also to induce microglial priming such that any subsequent insult exacerbates microglial inflammatory phenotype. Persistent activation of HPA axis with chronically high cortisol levels would compromise GR functions (Dejager et al., 2014). Further studies are needed to understand how GR activity is affected in microglia during chronically active HPA axis, as is the case for PD patients and whether GR inflammatory function is affected in PD. In addition, it would be important to understand the redundant and non-redundant functions of GR with closely related nuclear receptor members such as Nurr1 for envisaging therapeutic potentials in PD.

## Conflict of Interest Statement

The authors declare that the research was conducted in the absence of any commercial or financial relationships that could be construed as a potential conflict of interest.

## Abbreviations

ACTH, Adrenocorticotropic hormone; AP1, Activator protein 1; ATF, Activating transcription factor; BBB, Blood brain barrier; BST-1, Bone marrow stromal cell antigen; C1Q, Complement component 1q; C3, Complement component 3; CBP, CREB Binding protein; CCL5, Chemokine ligand 5; CD14, cluster of differentiation 14; CD200, Cluster of Differentiation 200; CD47/CD172A, Cluster of Differentiation 47; CLRN3, Clarin3; CNS, Central nervous system; CRH, Corticotropin-releasing hormone; CX3CL1, Chemokine (C-X3-C motif) ligand 1; CX3CR1, CX3C chemokine receptor 1; CYP2D6, Cytochrome P450 2D6; DAMP, Damage-associated molecular pattern; DBD, DNA-binding domain; ER-*β*, Estrogen Receptor *β*; FKBP51, FK506 binding protein; GC, Glucocorticoids; GPNMB, Glycoprotein NMB gene; GR, Glucocorticoid receptor; GRE, Glucocorticoid response element; H1N1, Hemagglutinin neuraminidase; HDAC2, Histone deacetylase 2; HLA, Human leucocyte antigen; HPA, Hypothalamic-pituitary-adrenal; HSP90, Heat shock protein 90; ICAM, Intercellular adhesion molecule; IFN-*γ*, Interferon *γ*; IκBa, Nuclear factor of κ light polypeptide gene enhancer in B-cells inhibitor; IKK, IκB kinase; IL-1*β*, Interleukin-1*β*; IL-6, Interleukin-6; iNOS, Inducible nitric oxide synthase; IRF, Interferon response factor; JNK, c-Jun N-terminal kinase; LB, Lewy bodies; LBD, Ligand-binding domain; LPS, Lipopolysaccharide; LRRK2, Leucine rich repeat kinase 2; LXR, Liver X receptor; MAPK, Mitogen-activated protein kinase; MCP1, Monocyte chemoattractant protein-1; MECP2, Methyl CpG binding protein 2; MHC, Major histocompatibility complex; MKP1, MAPK phosphatase 1; MMP9, Matrix metalloprotease 9; MPTP, 1-methyl, 4-phenyl, 1, 2, 3, 6-tetrahydropyridine; MR, Mineralocorticoid receptor; MSK1, Mitogen- and stress-activated protein kinase-1; MYD88, Myeloid differentiation primary response gene 88; NCOA1, Nuclear receptor coactivator 1; NF-κ*β*, Nuclear factor κ-light-chain-enhancer of activated *β* cells; NR3C1, Nuclear Receptor Subfamily 3, Group C, Member 1; NR4A, Nuclear receptor 4A; NSAID, Nonsteroidal anti-inflammatory drugs; P300, E1A binding protein p300; PAMP, Pathogen-associated molecular patterns; PD, Parkinson’s disease; PET, Positron emission tomography; PINK1, PTEN-induced putative kinase 1; PKA, Protein kinase A; PLA2G6, Phospholipase A2, group VI; PPAR-γ, Peroxisome proliferator-activated receptor *γ*; PVN, Paraventricular nucleus of hypothalamus; RANTES, Regulated on activation, normal T cell expressed and secreted- CCL5; REM, Rapid eye movement (REM) sleep; SN, Substantia nigra; SNCA, Synuclein, *α*; SNP, Single Nucleotide Polymorphism; SPG4/SPAST, Spastic paraplegia-4/spastin; TAK1, Transforming growth factor activated kinase-1; TLR, Toll-like receptor; TNF-*α*, Tumor necrosis factor-*α*.
